# Femur originated genu varum in a patient with symptomatic ACL deficiency: a case report and review of literature

**DOI:** 10.1186/s12891-021-04274-w

**Published:** 2021-05-13

**Authors:** Seyed Mohammad Javad Mortazavi, Abbas Noori, Farzad Vosoughi, Reza Rezaei Dogahe, Mohammad Javad Shariyate

**Affiliations:** Hip and Knee arthroplasty, Joint Reconstruction Research Center, Imam Khomeini hospital, Tehran University of Medical Sciences, End of Keshavarz Blvd, 1419733141 Tehran, Iran

**Keywords:** ACL reconstruction, Osteotomy, Knee arthroscopy

## Abstract

**Background:**

Anterior cruciate ligament (ACL) injury may be associated with genu varum. There are a few indications in which the varus deformity can be corrected at the time of ACL reconstruction. However, as the genu varum originates mostly from the tibia and the simultaneous presence of ACL deficiency and femur originated genu varum is uncommon, only a few papers have described their management for ACL deficient patients with femur originated genu varum.

**Case presentation:**

A young patient visited our clinic with a complaint of right knee pain and giving way. Further work up revealed a full mid substance ACL tear, mild medial knee osteoarthritis and femur originated genu varum of his right knee. He was managed with simultaneous ACL reconstruction and distal femoral valgus osteotomy.

**Conclusions:**

Any corrective osteotomy for genu varum should be performed at center of rotation angle. Isolated ACL reconstruction in patients with simultaneous ACL deficiency and genu varum may hasten the knee degeneration.

**Level of evidence:**

IV

## Background

Knee valgus osteotomy combined with Anterior Cruciate Ligament reconstruction (ACL-R) should be considered in active young patients with genu varum (varus angle > 5 degrees) and symptomatic ipsilateral Anterior Cruciate Ligament (ACL) deficiency if the affected knee has either medial compartment Osteoarthritis (OA) or lateral thrust (double varus) [1].

Among 650 cases of symptomatic ACL deficiency referred to our center during the three-years period (2016–2019), 8 patients had a genu varum plus either medial knee OA or lateral thrust and only one of them had femur originated genu varum. As the genu varum originates mostly from tibia and the simultaneous presence of ACL deficiency and femur originated genu varum is uncommon, only a few papers have described their management for ACL deficient patients with femur originated genu varum [2].

In this study, a young patient with symptomatic ACL deficiency, femur originated genu varum and mild medial OA of the right knee is reported. He was treated with simultaneous medial opening wedge distal femoral osteotomy (DFO) and ACL-R This report is presented according to the SCARE criteria as proposed by Agha et al. [3].

## Case presentation

Our patient was a 29-year-old man complaining of medial knee pain and giving way of his right knee for 2 years since a previous sport injury. His past medical history and drug history were negative.

During physical examination, the patient had no genu recurvatum. His Anterior Drawer Test (ADT) was positive with a 10 mm anterior subluxation. His Lachman and Pivot shift tests were positive. In addition to varus malalignment (varus angle 7 degrees), a lateral thrust was evident during his gait performance.

The Magnetic Resonance Imaging (MRI) confirmed ACL tear of his right knee. Our patient had double varus based on Noyes’s grading [1]. His Lysholm and International Knee Documentation Committee (IKDC) score were 26 and 29.8 respectively. The Visual Analogue Scale (VAS) score of his preoperative right knee pain was 8.

### Pre-operation planning

**Fig. 1:**
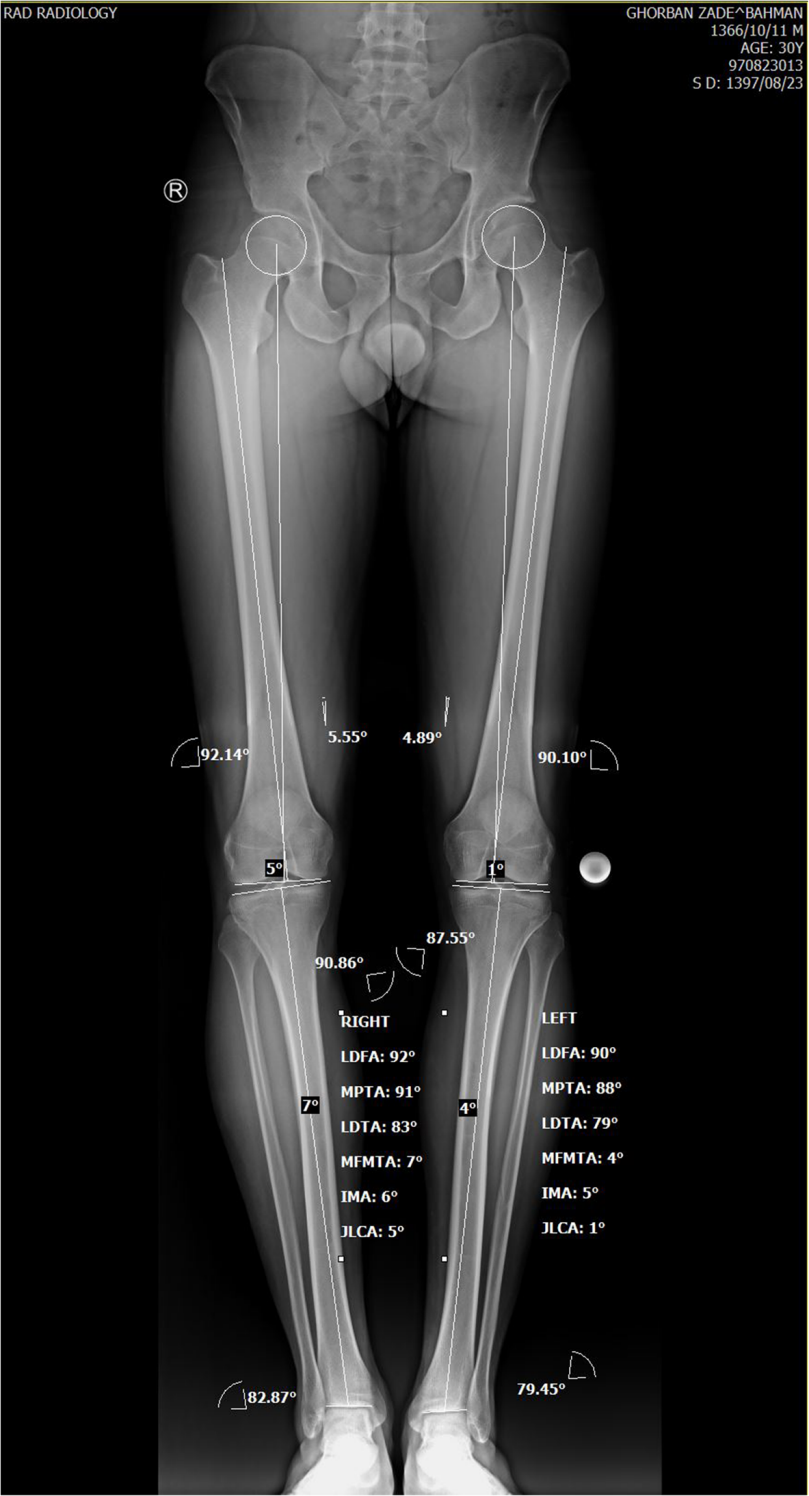
Pre-operative bilateral standing full length alignment views

Based on the patient’s standing triple joints alignment view, our patient’s varus angle was 7^o^ and according to his Medial Proximal Tibial Angle (MPTA) (90.83^o^) and Lateral Distal Femoral Angle (LDFA) (92.21^o^), the varus deformity was assumed to be of femoral origin (Fig. 1). So, it was decided to perform distal femoral valgus osteotomy to correct varus malalignment. No significant femoral/ tibial length discrepancy existed comparing both sides (femoral and tibial length of 492 and 413 mm on the right side consecutively; femoral and tibial length of 493 and 417 mm consecutively on the left side). Instead of lateral closing wedge osteotomy, it was preferred to perform medial side opening wedge valgus osteotomy as the former might interfere with femoral tunnel drilling during ACL-R. Using the mediCAD ® software, it was analyzed that at the osteotomy opening, the angle between proximal and distal fragments should be increased to 7 degrees and the space between the proximal and distal fragments should be increased to 10 mm at the medial cortex.

### Surgical technique

The patient was placed in a supine position. The involved lower limb was hung hyper-flexed with a leg holder. A prophylactic dose of Cefazolin (1 gram) and tranexamic acid (TXA) (1 gr) was administered intravenously 30 min and 1 h before the surgery consecutively. A same intravenous (IV) dose of TXA (1 gr) was repeated at the end of the surgery. A tourniquet was applied during the surgical procedure.

**Fig. 2:**
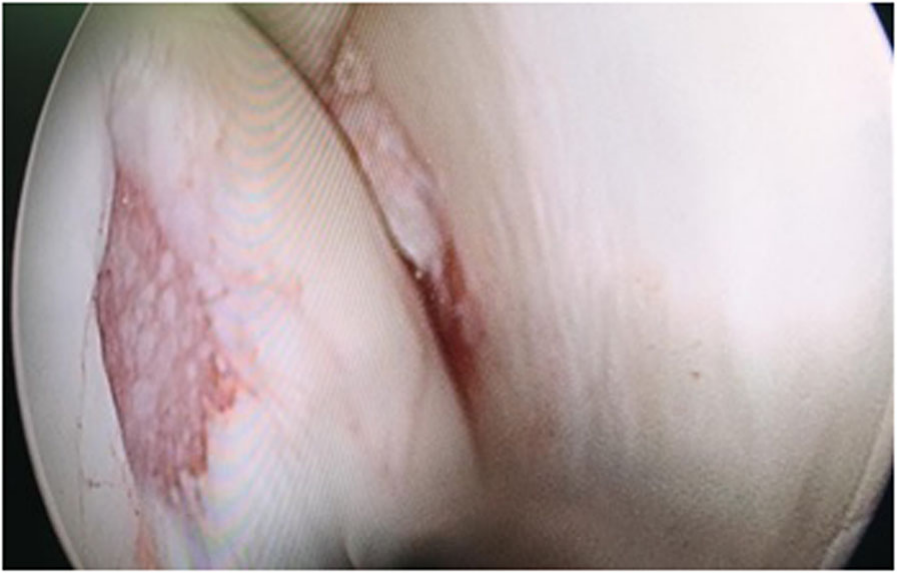
Chondral lesion in medial femoral condyle

**Fig. 3:**
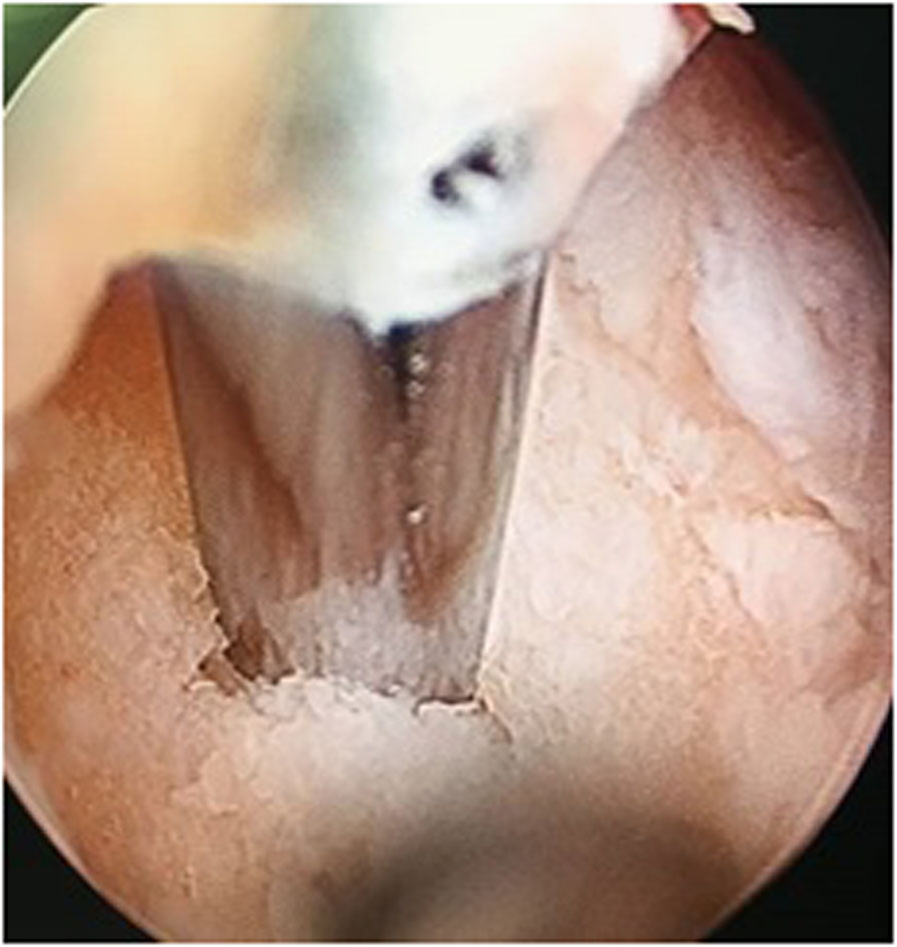
Creating micro fracture to stimulate the regeneration of the chondral lesion in the medial femoral condyle

Arthroscopy was performed through standard anteromedial and anterolateral portal. A mid substance tear in ACL plus 1cm^2^ chondral lesion in medial femoral condyle were diagnosed (Fig. 2). The stump of the ACL was removed. Then, chondral abrasion and micro fracturing was performed to stimulate the regeneration of chondral lesion (Fig. 3). No meniscal lesion was detected.

### Graft harvesting

A 3 cm vertical incision was made on the anteromedial tibial cortex initiating at 1 cm distal to the joint line of the knee. The sartorial fascia was dissected and the Semitendinous and Gracilis tendons were recognized. All vincula of both tendons were released carefully and tendon stripper was then used to harvest Gracilis and Semitendinous autograft.

**Fig. 4:**
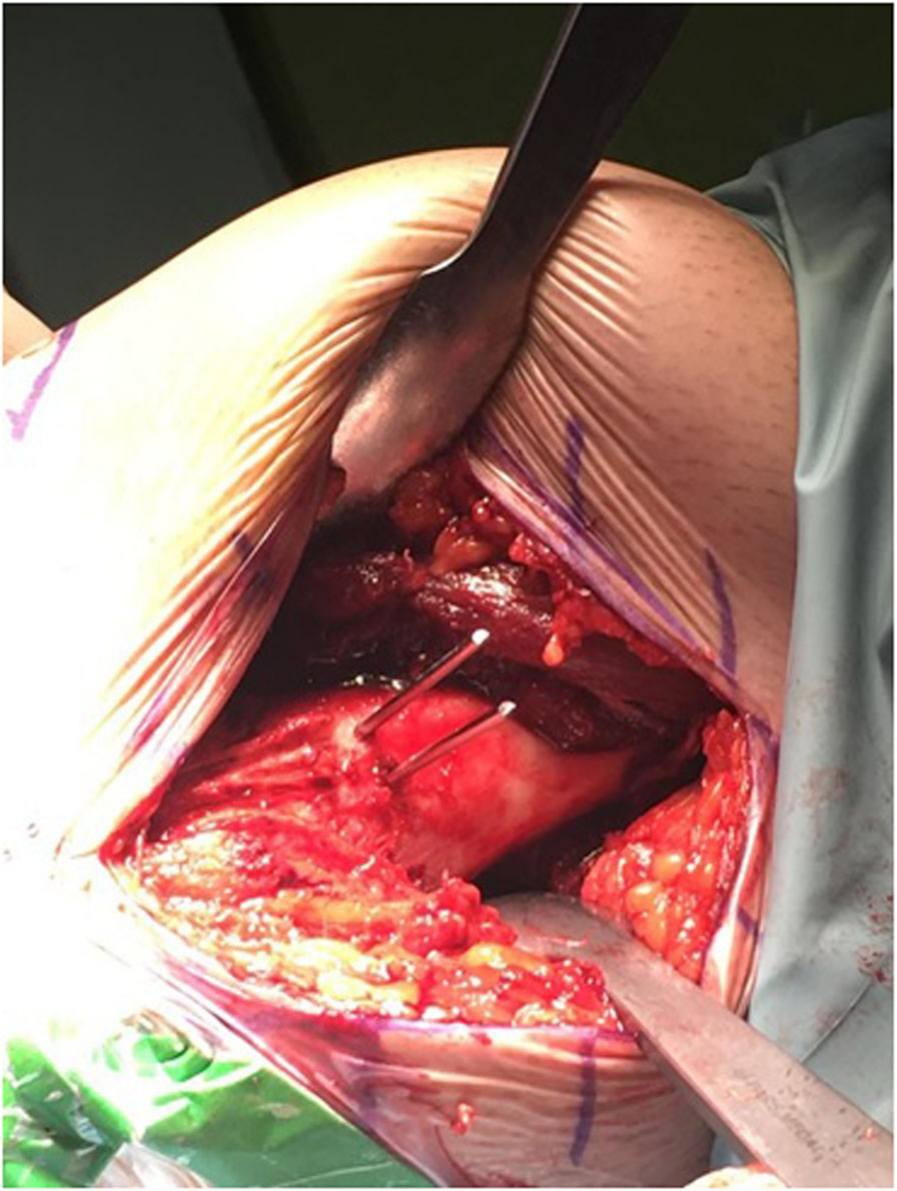
Two parallel pins were inserted before performing osteotomy from 55 millimeters proximal to the knee joint to just proximal to the lateral knee condyle

After diagnostic arthroscopy, the knee was extended and the leg was placed on a Mayo table. A longitudinal incision on anteromedial side of the right thigh was created starting from 10 cm above the upper border of the patella to 2 cm bellow the patella’s upper border. The distal femur was exposed through subvastus approach. The joint capsule was left intact. Under fluoroscopic guidance, two pins were placed from 55 mm proximal to the joint line on the medial side to just proximal to the lateral femoral condyle. The first pin was inserted at the intersection of anterior one third and posterior two thirds of the femur and the second one was applied in the intersection of posterior one third and anterior two thirds of the femur in parallel to the first one (Fig. 4).

**Fig. 5:**
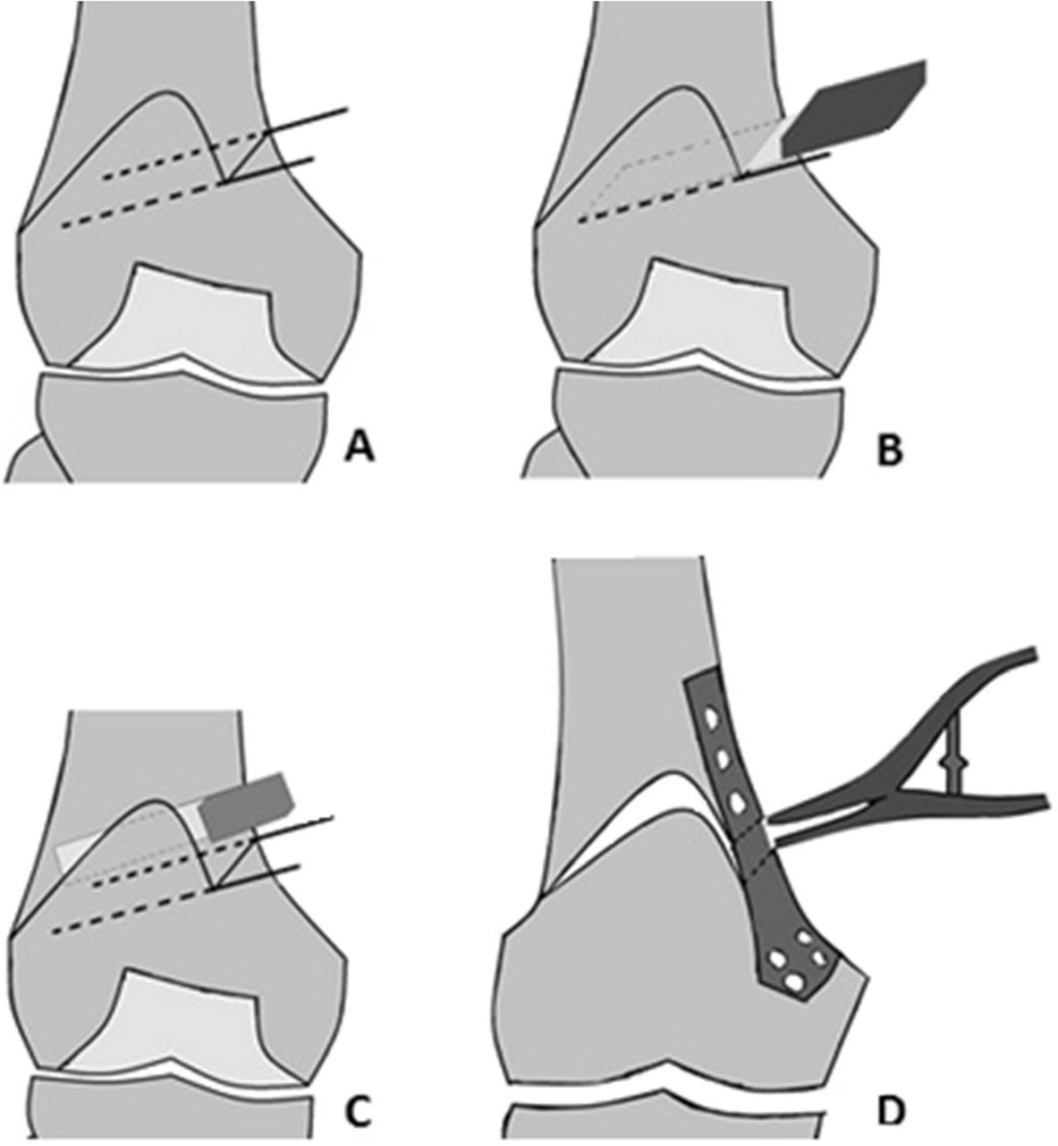
Different stages of the medial open wedge distal femoral osteotomy. From 55 millimeters proximal to the knee joint to just proximal to the lateral knee condyle, two pins are inserted (**a**). Osteotomy in the posterior three fourth of the Femur is made parallel to the inserted pins in a way that 1 centimeter near the lateral cortex remains intact (**b**). The ventral one fourth of the femur is cut vertically (**c**). After completing the osteotomy, the gap between the proximal and distal femur is increased to reach the scheduled angle between the two parts. Then the TomoFix plate is placed in order to fix the fracture site and the gap is filled with bone graft (**d**)

Biplanar osteotomy was performed from medial to lateral direction (Fig. 5). Protecting the soft tissue dorsally with a Hohmann retractor and constantly cooling the oscillating saw blade, a transverse osteotomy cuts was performed in the posterior three fourth of the femur parallel to the path of the inserted pins. In the ventral one fourth of the femur, the osteotomy was performed vertically according to the biplanar osteotomy technique. The vertical osteotomy cut was accomplished with a thinner saw blade to prevent further soft tissue injuries. Posterior cortex was cut with manual broad osteotome to protect the neurovascular structures. During the osteotomy, one centimeter of the cortex on the lateral side was kept intact to function as a hinge during the wedge insertion.

Careful opening of osteotomy site was performed gradually with variable sized chisel osteotome to save the lateral femoral cortex. Lamina spreader was placed to insert the wedge shape corticocancellus allograft.

**Fig. 6:**
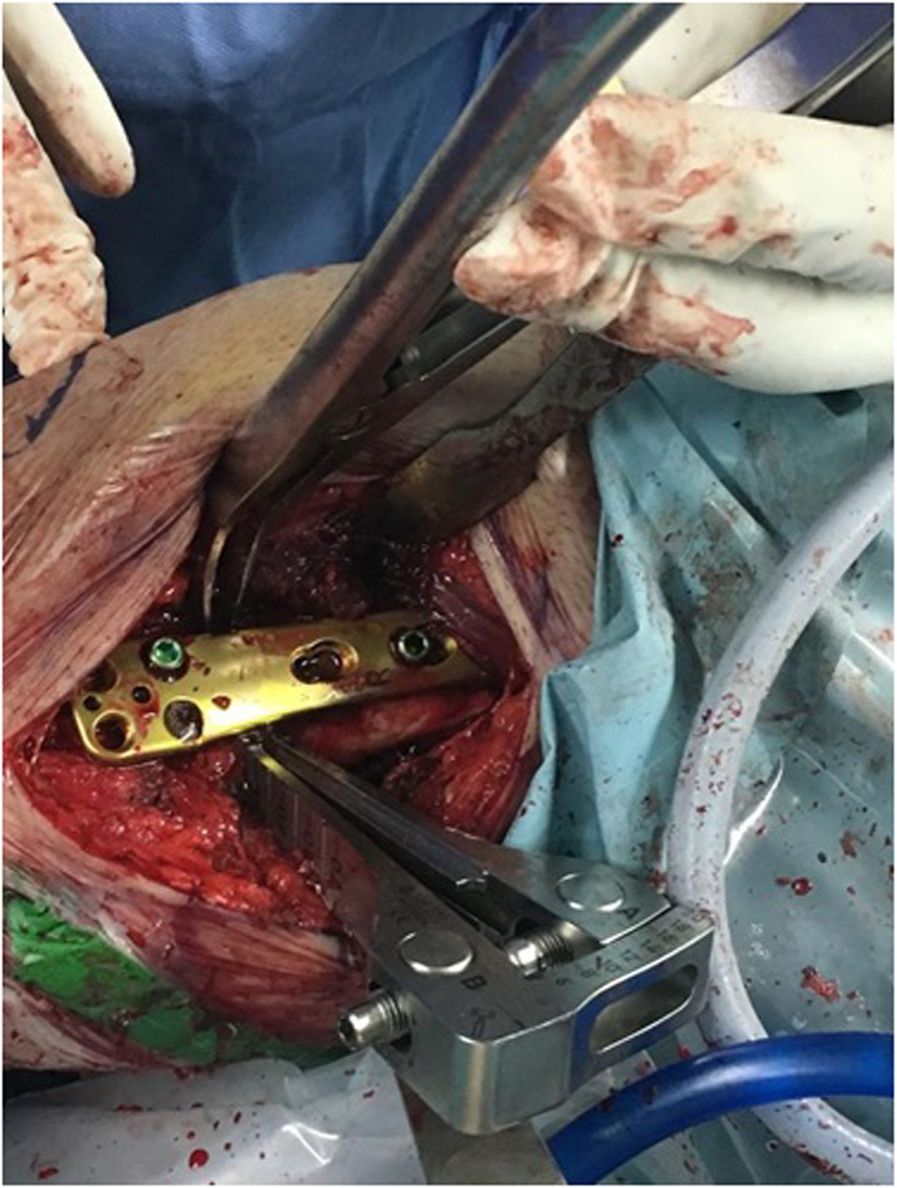
Provisonal plate fixation before femoral canal drilling

The osteotomy was stabilized with TomoFix Medial Distal Femur Plate (Synthes®) placed anteromedially (Fig. 6). Four bicortical screws were applied at the proximal segment and 4 unicortical screws were used at the distal fragment in such a way not crossing the midline.

In the next step, the leg was hung on the leg holder again and the femoral and tibial canals were created arthroscopically. The harvested hamstring autograft was passed through the tibial and femoral tunnel. It was fixed proximally by flipping an endo-button on the lateral femoral cortex and distally to the tibia by interference bioabsorbable screw. After careful hemostasis and wound irrigation with sterile normal saline, the surgical incision was repaired without placing any drainage catheter.

### Post-operative management

Following the surgery, the patient was allowed to start range of motion. Partial weight bearing in a hinge knee brace was permitted during the first 6 weeks postoperatively. After 6 weeks, as the clinical and radiologic signs of bone union were observed, the patient was allowed to progress to a full weight bearing status and discontinue the brace. Closed chain exercise was practiced during the first 6 weeks and then, the open kinematic chain exercise was allowed.

**Fig. 7:**
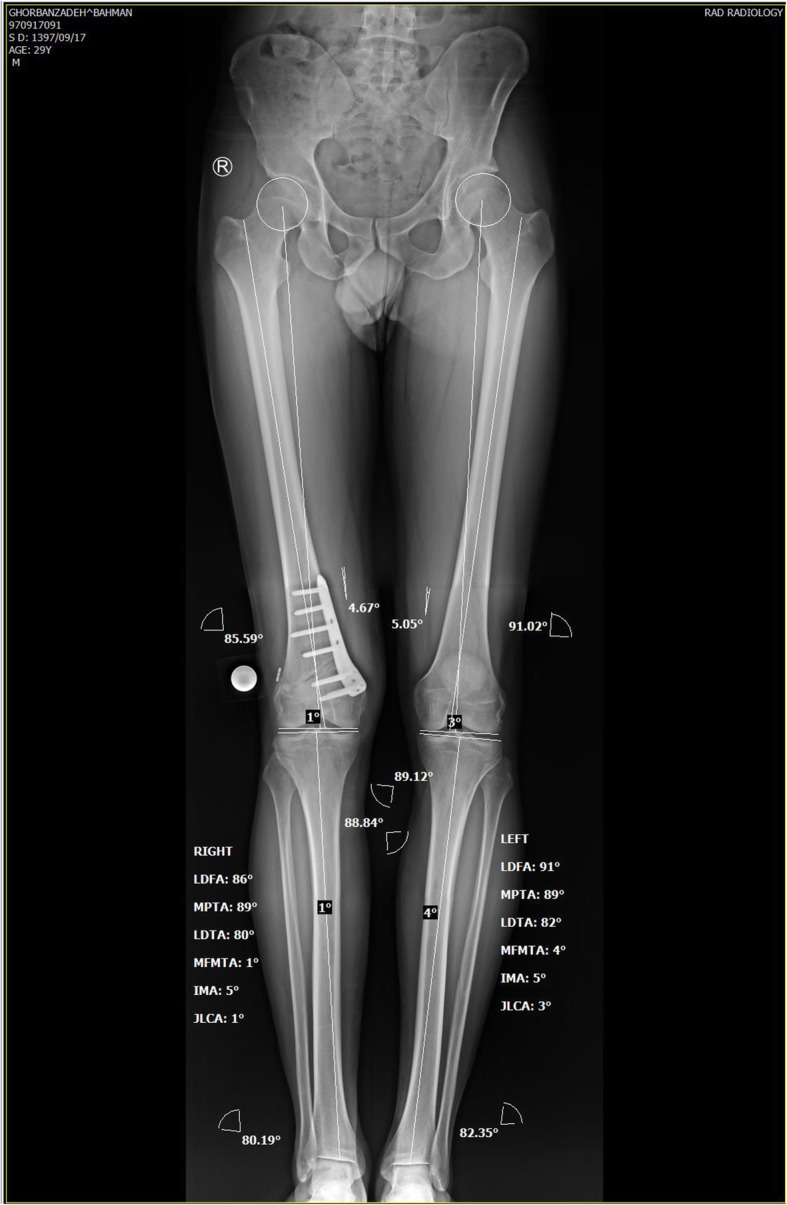
Postoperative bilateral standing full length alignment view

Long standing alignment view, at 12 weeks postoperatively, confirmed deformity correction (Fig. 7). The valgus angle was analyzed to be 2^o^. the detailed alignment variables before and after surgery are depicted in Table 1.

**Table 1:** The preoperative and postoperative knee alignment variables based on the triple joints standing views

MPTA	90.83	89
LDFA	92.21	85
VA	7	-2
JLCA	5	1
PS	12.1	12.1

Abbreviations: *MPTA* medial proximal tibial angle; *LDFA* lateral distal femoral angle; *VA* varus angle; *JLCA* joint line congruence angle; *PS* posterior slope of the tibia.

After 6 months, complete union was achieved and our patient’s Lachman and pivot tests were negative. The patient reported no pain during his daily activities (VAS = 0). The amount of anterior subluxation in the ADT was 3mm. The Lysholm and IKDC score were 99 and 94.4 respectively.

## Discussion

The knee with an ACL deficiency presents several problems. Episodes of giving way due to anterior knee instability could result in meniscal tears and degeneration of the articular cartilage [4]. Curado et al. demonstrated that moderate to severe knee OA affects 29 % of ACL deficient patients during a period of 22 years [5].

Long lasting genu varum imposes greater force on the medial knee compartment, which could cause osteoarthritis as well as degeneration of the articular cartilage [4].

Dejour et al. demonstrated that isolated ACL reconstruction in ACL deficient knees with accompanying chondral or meniscal injury can paradoxically accelerate the process of OA and result in earlier reoperation [6]. As ACL reconstruction restores the knee stability, it motivates the patient to return to activity and sport faster (permission to abuse the knee). Thus, this might hasten the knee degeneration if the osteoarthritic etiologies (i.e. genu varum, meniscal and knee chondral injury, etc.) is not considered to address simultaneously. While ACL reconstruction stabilizes the knee joint, the valgus osteotomy improves medial knee pain caused by the genu varum, prevents further knee degeneration and decreases the tension on the ACL [4, 7].

Therefore, in those with symptomatic ACL deficiency and medial knee OA due to a varus knee, staged or simultaneous ACL reconstruction and valgus osteotomy is recommended. As simultaneous management of both ACL deficiency and varus deformity in a single operation results in a less recovery time and comparable operative complication than the 2-stage operation, simultaneous ACL-R and valgus osteotomy may be a viable option especially in active young athletic patients [7–10].

Li et al. performed a systematic review and concluded that ACL-R simultaneously performed with HTO restores the anterior stability of the knee, prevents further advancement of medial knee OA and return patients to sport activity [11].

Different studies evaluated the return to sport after simultaneous ACL-R and high tibial valgus osteotomy. Bonin et al. demonstrated that around 47 % of patients return to sport activity during a period of 12 years [12]. In comparison, Schneider et al. followed his patients for 10 years and observed 80 % of patients return to sport, while around 30 % gain their preinjury sport activity level [13].

Despite describing and evaluating simultaneous HTO and ACL-R by many studies, [4, 12–15], only a few have described simultaneous ACL-R and DFO [2]. In our case, as the deformity had femoral origin (MPTA = 90, LDFA = 92), the valgus osteotomy was carried out in the distal femur.

Different studies revealed that open wedge HTO is associated with increase in posterior tibial slope [16–20]. As posterior tibial slope increases by 10 units, anterior tibial translation increases by 6 millimeters leading to more tension on the ACL [21]. Compared to simultaneous ACL reconstruction and opening wedge HTO, combined ACL reconstruction and DFO does not affect the posterior tibial slope and may have a possible advantage of correcting the knee alignment without increasing the posterior tibial slope.

Regarding our patient, no significant femoral/tibial length discrepancy existed comparing both sides (femoral and tibial length of 492 and 413 mm on the right; femoral and tibial length of 493 and 417 mm consecutively on the left side). Due to this fact and the concern of possible interference of lateral closing wedge DFO and femoral tunnel drilling during ACL reconstruction, in contrast to Moradi et al. study [2], it was decided to perform a medial opening wedge DFO. After the surgery, the varus angle and the LDFA of the affected knee decreased to the normal level (valgus angle 2, LDFA 85). The MPTA of the same side seems to be decreased from 90.83 to 89. However, as no corrective alignment procedure was performed on the tibia, we believe the nearly 1 degree change in MPTA to be a measurement error. Further studies however, may be needed to compare the results of simultaneous ACL-R with medial opening wedge versus lateral closing wedge DFO, their impact on quality of life and return to sport to further reveal the benefits and limitations of the procedure.

In conclusion, this case report emphasizes that any corrective osteotomy for genu varum should be performed at center of rotation angle. Isolated ACL-R in patients having ACL deficiency and genu varum may lead to increased usage of the affected knee and exacerbate knee degenerative joint disease.

## Data Availability

not applicable.

## Abbreviations

ACL: Anterior cruciate ligament
ACL-R: Anterior cruciate ligament reconstruction
OA: Osteoarthritis
SCARE: Surgical case report
ADT: Anterior drawer test
MRI: Magnetic resonance imaging
IKDC: International Knee Documentation Committee
VAS: Visual analogue scale
MPTA: Medial proximal tibial angle
LDFA: Lateral distal femoral angle
JLCA: Joint line congruence angle
VA: Varus angle
PS: Proximal tibial posterior slope
IV: Intravenous
TXA: Tranexamic acid
mm: Millimeter
cm: Centimeter
cm^2^: Cubic centimeter
HTO: High tibial osteotomy
DFO: Distal femoral osteotomy
